# Supplementary material for: KLK6/PAR1 Axis Promotes Tumor Growth and Metastasis by Regulating Cross-Talk between Tumor Cells and Macrophages
Source: Cells. 2022 Dec 16;11(24):4101. doi: 10.3390/cells11244101 (PMC9777288; doi:10.3390/cells11244101)
Supplement: Supplementary file 1 [file cells-11-04101-s001.zip › cells-1984979-supplementary.pdf]

Supplementary Figure S1

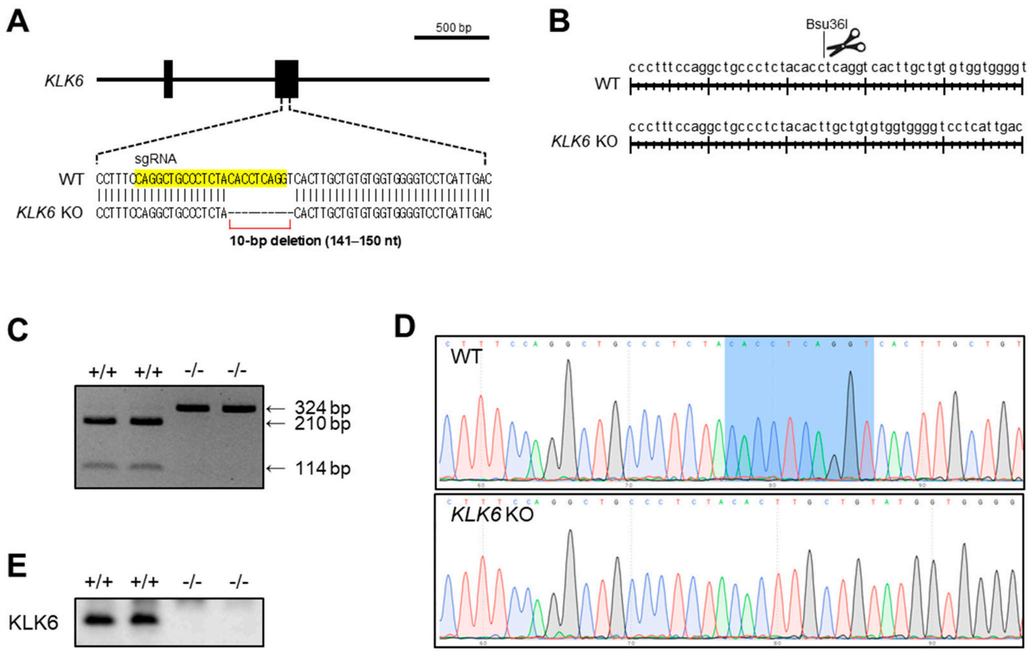

Supplementary Figure S1. Generation and characterization of KLK6-/- mice. (A) Schematic diagram of sgRNA-targeting sites in KLK6 gene. The sgRNA-targeting sequence is labeled in yellow. The deletion site of KLK6-deficient mice is shown as red. (B) Schematic diagram of the Bsu36I enzyme site, which can cut to the deleted part of the sequence of the wild type (WT) KLK6 gene but cannot cut the KLK6 gene of KLK6-deficient mouse. (C) PCR genotyping with a restriction enzyme. Genomic DNA samples from the WT and KLK6-deficient mice were amplified by PCR. PCR products were cut using the Bsu36I enzyme. (D) Representative sequence of KLK6 from WT and KLK6-deficient mouse. The deleted nucleotides are indicated using blue. (E) Immunoblotting results for KLK6 protein expression levels in WT and KLK6-deficient mice serum; +/+, wild-type; −/−, homozygous knockout.

Supplementary Figure S2

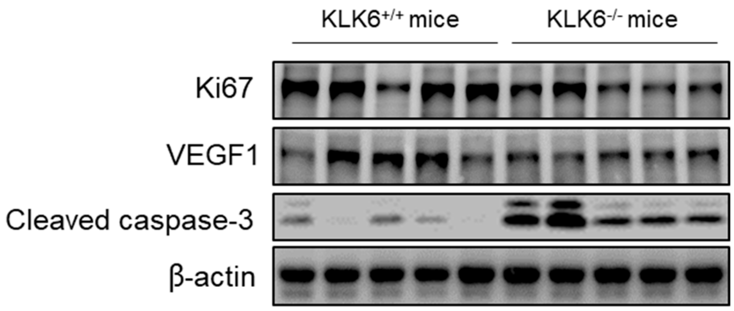

Supplementary Figure S2. The expression of Ki67, VEGF1, and cleaved caspase-3 in tumor tissues. 1 × 10^6 cells of B10F10 melanoma were subcutaneously injected into WT and KLK6−/− mice. After two weeks, tissue samples were collected from primary tumors. The levels of Ki67, VEGF1, and cleaved caspase-3 proteins in primary tumor tissues from WT and KLK6−/− mice were analyzed by Western blot. Data are representative of two experiments. +/+, wild-type; −/−, homozygous knockout.

Supplementary Figure S3

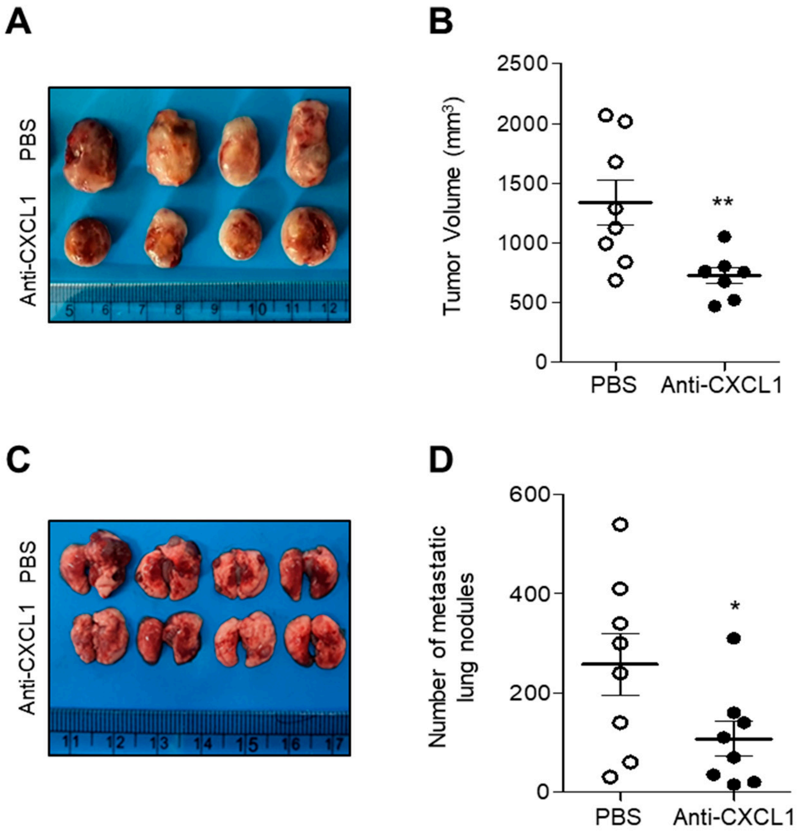

Supplementary Figure S3. CXCL1 inhibition decreased for tumor growth and metastasis of LLC cells. (A and B) LLC cells (1×10^6) were subcutaneously injected into WT mice (n = 8 per group). After 1 h, mice were injected with PBS (control) or CXCL1 neutralizing antibody (5 mg/kg) every other day for 10 days. (A) Representative photograph of the tumors. (B) Tumor volume was calculated as described in Materials and Methods. (C and D) LLC cells (1×10^6) were intravenously injected into the tail vein of WT mice (n = 8 per group). After 1 h, the mice were injected with PBS (control) or CXCL1 neutralizing antibody (5 mg/kg) every other day for 10 days. (C) Representative photograph of metastatic lung nodules. (D) The number of metastatic lung nodules was calculated as described in Materials and Methods. **P < 0.01 and * P < 0.05 (Student's t-test). +/+, wild-type; −/−, homozygous knockout.

Supplementary Figure S4

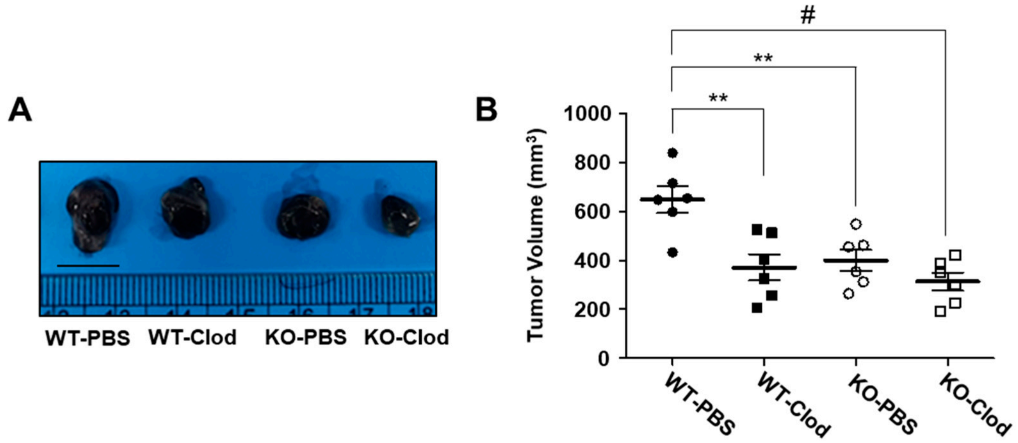

Supplementary Figure S4. The effect of macrophage depletion on tumor growth. 1 × 10^6 cells of B16F10 were subcutaneously injected into WT and KLK6−/− mice (n=6 per group). After tumors were established (5 days post-injection), PBS-liposome or clodronate-liposome were injected IV every five days until the end of the experiment. (A) Representative photograph of tumors. (B) Tumor volume was calculated as described in Materials and Methods. **p<0.01, #p<0.001. WT, wild-type; KO, KLK6 knockout.

Supplementary Figure S5

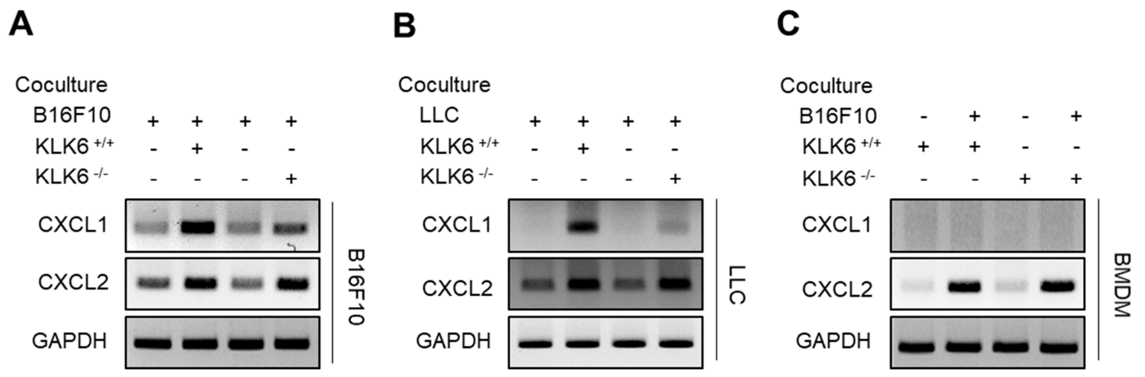

Supplementary Figure S5. CXCL1 was expressed in B16F10 cancer cells but not in BMDMs. B16F10 and LLC cells were cocultured with WT or KLK6-/- BMDMs for 24 h. The mRNA levels of CXCL1 and CXCL2 in B16F10 cells (A), LLC cells (B), and BMDMs (C) were analyzed by RT-PCR. +/+, wild-type; −/−, homozygous knockout.

Supplementary Figure S6

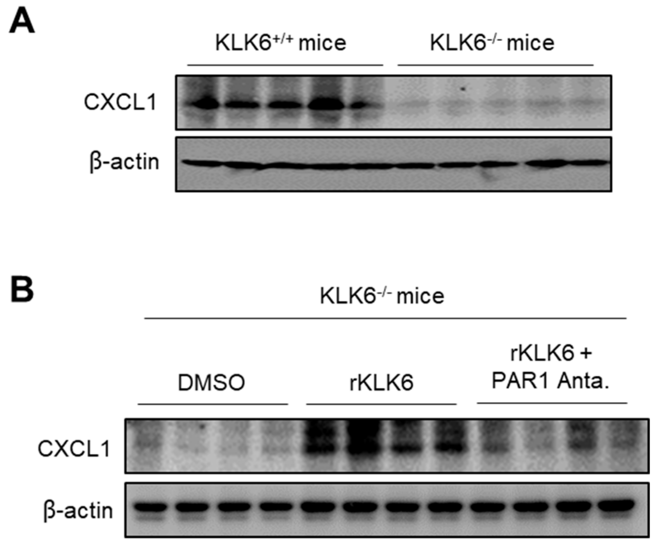

Supplementary Figure S6. The expression of CXCL1 in tumor tissues. (A) 1×10^6 cells of B10F10 melanoma were subcutaneously injected into WT and KLK6−/− mice. (B) 1×10^6 cells of B10F10 cells were subcutaneously injected into KLK6-/- mice. After 1 h, DMSO (control), rKLK6 (10 mg/kg), or rKLK6 plus PAR1 (5 mM/kg) were injected every other day for ten days. After two weeks, tissue samples were collected from primary tumors. The level of CXCL1 proteins in primary tumor tissues from WT and KLK6−/− mice were analyzed by Western blot. +/+, wild-type; −/−, homozygous knockout.

Supplementary Figure S7

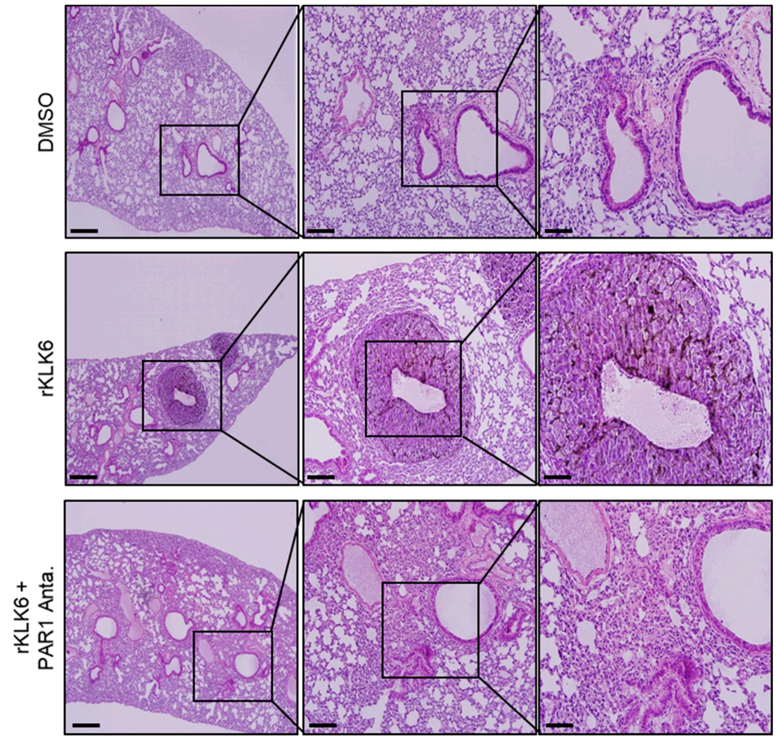

Supplementary Figure S7. PAR1 inhibitor reduced rKLK6-mediated lung metastasis. 5×10^5 cells of B16F10 melanoma were injected into the tail vein of KLK6 -/- mice. After 1 h, mice were injected with DMSO (control), rKLK6 (10 mg/kg), or rKLK6 plus PAR1 (5 mM/kg) on alternate days for 10 days. Sections of lung organs excised from mice were stained with H&E.

Supplementary Figure S8

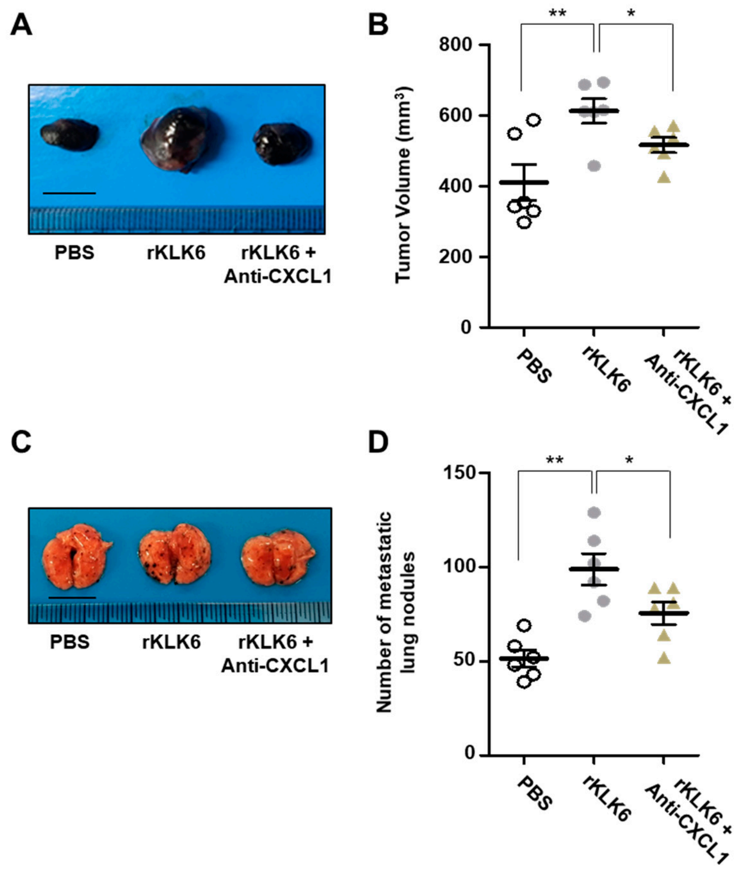

Supplementary Figure S8. The effect of CXCL1 inhibition on rKLK6-mediated tumor growth and lung metastasis. (A and B) B16F10 cells (1 × 10^6) were subcutaneously injected into KLK6-/- mice. After 1 h, mice were injected with DMSO (control), rKLK6 (10 mg/kg), or rKLK6 plus CXCL1-neutralizing antibody (5 mg/kg) on alternate days for ten days. (A) Representative photograph of the tumors. (B) The tumor volume was measured as described in Materials and Methods. (C and D) B16F10 cells (5 × 10^5) were injected into the tail vein of WT mice. mice were injected with DMSO (control), rKLK6 (10 mg/kg), or rKLK6 plus CXCL1-neutralizing antibody (5 mg/kg) on alternate days for ten days. (C) Representative photograph of metastatic lung nodules. (D) The number of metastatic lung nodules was calculated as described in Materials and Methods. **$P < 0.01$ and * $P < 0.05$ (Student's t-test).

Supplementary Figure S9

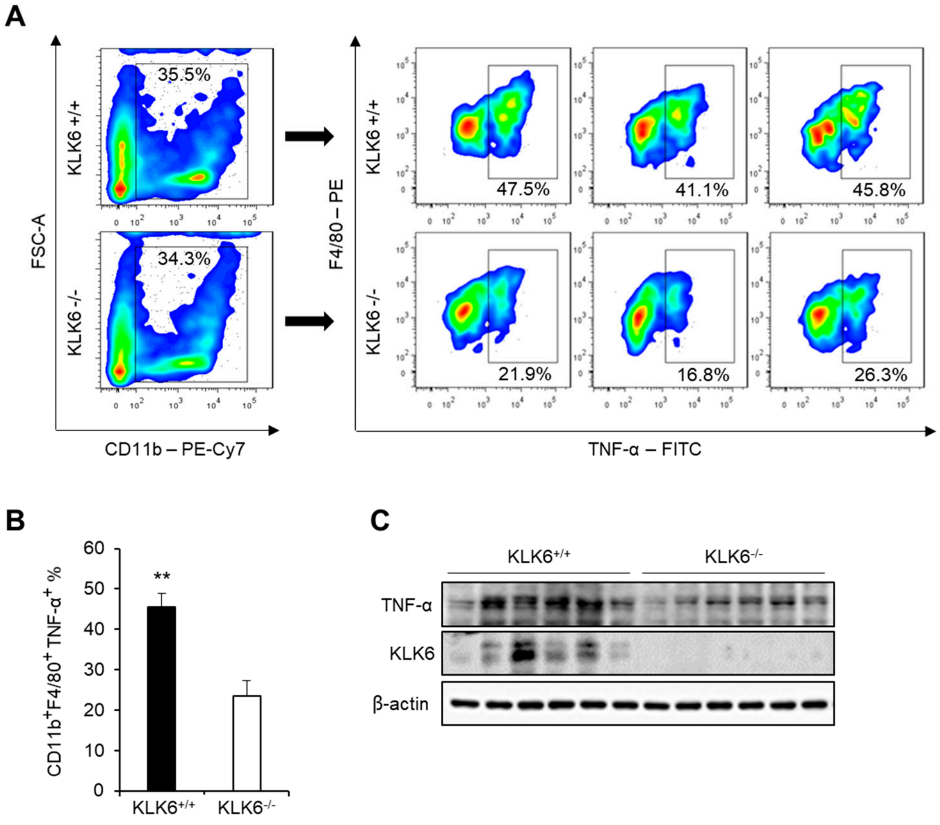

Supplementary Figure S9. TNF-*α* were dominantly expressed in macrophages from tumor tissues of WT mice. B10F10 cells (1 × 10^6) were subcutaneously injected into WT and KLK6−/− mice. Tumor tissue samples were prepared as described in Materials and Methods (n = 6 per group). CD11b+/F4/80+ macrophage cells were isolated from tumor tissues as described in Materials and Methods. (A) Flow-cytometry analysis of intracellular TNF-*α* expression in CD11b+/F4/80+ macrophage cells from WT and KLK6−/− mice tumor tissues. (B) Quantified graph showing the percentage of macrophages with CD11b+/F4/80+/TNF-*α*+ phenotype in tumor tissues. (C) The levels of TNF-*α* and KLK6 protein in macrophages isolated from tumor tissues of WT and KLK6−/− mice were analyzed by Western blot. +/+, wild-type; −/−, homozygous knockout. **P < 0.01 (Student's t-test).

Table S1

Table S1. Sequences of siRNA oligonucleotide template.

siRNA	(5') Sense Sequences (3')	(5') Antisense Sequences (3')
KLK6 #1	CUCUACACCUCAGGUCACUdTdT	AGUGACCUGAGGUGUAGAGdTdT
KLK6 #2	GGUGUGGUUAGCAGAUGGAdTdT	UCCAUCUGCUAACCACACCdTdT
PAR1 #1	GACGAGAGGAGAAAGGUGUdTdT	ACACCUUUCUCCUCUCGUCdTdT
PAR1 #2	GUGUUCGUCUUGAGGAUGAdTdT	UCAUCCUCAAGACGAACACdTdT
PAR2 #1	AGAUCUCCUACCACCUCAAdTdT	UGUAGGUGGUAGGAGAUCUdTdT
PAR2 #2	CACUGUGAAUCGCAUGCAAdTdT	UUGCAUGCGAUUCACAGUGdTdT

Table S2

Table S2. DNA oligomers list used in RT-PCR.

Gene Name	(5') Forward Primers (3')	(5') Reverse Primers (3')
KLK6	GCCCTCTACACCTCAGGTCA	ATCACCTGCAGATTCGGTTT
PAR1	GTTGATCGTTTCCACGGTCT	GCAGACGATGAAGATGCAGA
PAR2	AACATCACCACCTGTCACGA	GCACGTAGGCAGATGCAGTA
CXCL1	CTTGAAGGTGTTGCCCTCAG	TGGGGACACCTTTTAGCATC
CXCL2	AGTGAACTGCGCTGTCAATG	GCCCTTGAGAGTGGCTATGA
GAPDH	TGTTCCTACCCCCAATGTGT	CCCTGTTGCTGTAGCCGTAT
